# Improved anticancer efficacy of methyl pyropheophorbide-a–incorporated solid lipid nanoparticles in photodynamic therapy

**DOI:** 10.1038/s41598-023-34265-x

**Published:** 2023-05-06

**Authors:** Sooho Yeo, Tae Heon Lee, Min Je Kim, Young Key Shim, Il Yoon, Young Kyu Song, Woo Kyoung Lee

**Affiliations:** 1 Center for Nano Manufacturing and Department of Nanoscience and Engineering, Inje University, Gimhae, 50834 South Korea; 2 Present Address: Yonsei Institute of Pharmaceutical Sciences, College of Pharmacy, Yonsei University, Seoul, South Korea; 3 Research Center of Dr. I&B Co., DaeJeon, Republic of Korea

**Keywords:** Cancer, Chemistry, Nanoscience and technology

## Abstract

Photodynamic therapy (PDT) is a promising anticancer treatment because it is patient-friendly and non-invasive. Methyl pyropheophorbide-a (MPPa), one of the chlorin class photosensitizers, is a drug with poor aqueous solubility. The purpose of this study was to synthesize MPPa and develop MPPa-loaded solid lipid nanoparticles (SLNs) with improved solubility and PDT efficacy. The synthesized MPPa was confirmed ^1^H nuclear magnetic resonance (^1^H-NMR) spectroscopy and UV–Vis spectroscopy. MPPa was encapsulated in SLN via a hot homogenization with sonication. Particle characterization was performed using particle size and zeta potential measurements. The pharmacological effect of MPPa was evaluated using the 1,3-diphenylisobenzofuran (DPBF) assay and anti-cancer effect against HeLa and A549 cell lines. The particle size and zeta potential ranged from 231.37 to 424.07 nm and − 17.37 to − 24.20 mV, respectively. MPPa showed sustained release from MPPa-loaded SLNs. All formulations improved the photostability of MPPa. The DPBF assay showed that SLNs enhanced the ^1^O_2_ generation from MPPa. In the photocytotoxicity analysis, MPPa-loaded SLNs demonstrated cytotoxicity upon photoirradiation but not in the dark. The PDT efficacy of MPPa improved following its entrapment in SLNs. This observation suggests that MPPa-loaded SLNs are suitable for the enhanced permeability and retention effect. Together, these results demonstrate that the developed MPPa-loaded SLNs are promising candidates for cancer treatment using PDT.

## Introduction

Photodynamic therapy (PDT) is one of the anticancer treatment strategies that is patient-friendly, noninvasive, and highly localized^1^. A photosensitizer (PS) essentially is a PDT drug that exerts pharmacological effects in the presence of light of an appropriate wavelength. Upon irradiation with light, the PS generates reactive oxygen species, particularly singlet oxygen (^1^O_2_), that triggers cancer cell death^1–3^. PS are preferentially localized in tumor sites that are harmless to adjacent healthy cells^4,5^. PDT offers the advantages of extremely low systemic toxicity and excellent function-sparing treatment as compared to conventional cancer treatment strategies, including chemotherapy, surgery, radiotherapy, and immunotherapy^1,2,6–8^.

Researchers continue to search for an ideal PS that interacts with light of a relatively long wavelength. Light with long wavelengths effectively penetrates the human body and can treat deeply placed tumors^2,9,10^. Methyl pyropheophorbide-a (MPPa), one of the chlorin class of PSs, is a second-generation PS with relatively long wavelength absorption as compared with the porphyrin class of the first-generation PS^11^. The molecular weight of MPPa is 548.7 g/mol.

MPPa has relatively high hydrophobicity, leading to low bioavailability, as it suffers significantly from administration challenges^2,7,9,12^. Various approaches have been proposed to enhance the solubilization of poorly water-soluble drugs, including conjugation of PS to water-soluble polymers^13^, entrapment of PS into polymer-based particles^9,14^, amorphization^9,15^ and nanonization^2,9,16^. However, considering the time-consuming amorphization process and the concerns of storage stability attributed to the hygroscopic properties of polymers, it is important to investigate pharmaceutical technologies that could enhance the solubility of MPPa^9,17^.

Solid lipid nanoparticle (SLN) is a promising drug delivery system for enhancing aqueous solubility and stability of poorly water-soluble drugs^2,7,9,18,19^. In the SLN system, a hydrophobic drug is molecularly dispersed in a solid lipid matrix as an oil (O) phase, and the drug-dissolved O phase is then stably dispersed in water by forming an oil-in-water (O/W) phase^9,18,20^. The solid lipid of the SLN system physically protects the entrapped drug from external environments (light, pH, and atmospheric moisture)^21–24^. In cancer therapy, conventional pharmaceutical strategies have used the enhanced permeability and retention (EPR) effect for passive targeting of agents^25,26^. Anticancer drugs up to a size of 400 nm can spontaneously accumulate in tumors with leaky vasculature^27–29^.

We hypothesized that SLN can improve the solubility and pharmacological effects of MPPa based on the EPR effect used itn cancer therapy. We synthesized MPPa from chlorophyll and fabricated the MPPa-loaded SLN. The structure of the synthesized MPPa was confirmed using ^1^H nuclear magnetic resonance (^1^H-NMR) spectroscopy, and the pharmaceutical characteristics of MPPa-loaded SLNs were determined using particle characteristics and Fourier transform infrared spectroscopy (FTIR). We assessed the PDT efficacy by analyzing ^1^O_2_ production using 1,3-diphenylisobenzofuran (DPBF) as a non-biological assay and by evaluating the viability of two cancer cell lines (HeLa from human cervical carcinoma and A549 from human lung carcinoma) using the WST biological assay.

## Materials and methods

### Materials

Phosphate-buffered saline (PBS), methylene blue (MB), and chloroform were purchased from Sigma-Aldrich (St. Louis, MO, USA). Palmitic acid (PA) and stearic acid (SA) were supplied by SAMCHUN (Pyeongtaek, Korea). Glycerol monostearate (GMS) was obtained from Kanto Chemical Co, Japan. Inc. (Tokyo, Japan), and Poloxamers (PX) 188 and PX 407 were procured from BASF (Ludwigshafen, Germany). Tween 80 (TW 80) was purchased from Dae Jung Co., Ltd. (Busan, Korea) and chlorophyll-a paste from Shandong Lanmo Biotech Co. Ltd. (Shanghai, China). Methylene chloride (CH_2_Cl_2_, MC) and DPBF were supplied by Duksan Co. Ltd. (Gyeonggi-do, Korea) and TCI Chemicals (Tokyo, Japan), respectively. Dulbecco’s modified Eagle’s medium (DMEM) was provided by WelGENE (Gyeongsan, South Korea), and penicillin–streptomycin solution (100 ×) and fetal bovine serum (FBS) were purchased from BioWest (Nuaillé, France). The cancer cell lines (HeLa and A549) were obtained from the Korea Cell Line Bank (Seoul, Korea), and the Quanti-MAX WST-8 assay kit from Biomax (Seoul, Republic of Korea). High-performance liquid chromatography (HPLC)-grade methanol (MeOH) was purchased from Honeywell (Seelze, Germany). All other chemicals used were of HPLC grade.

### Synthesis of MPPa

**Figure 1:**
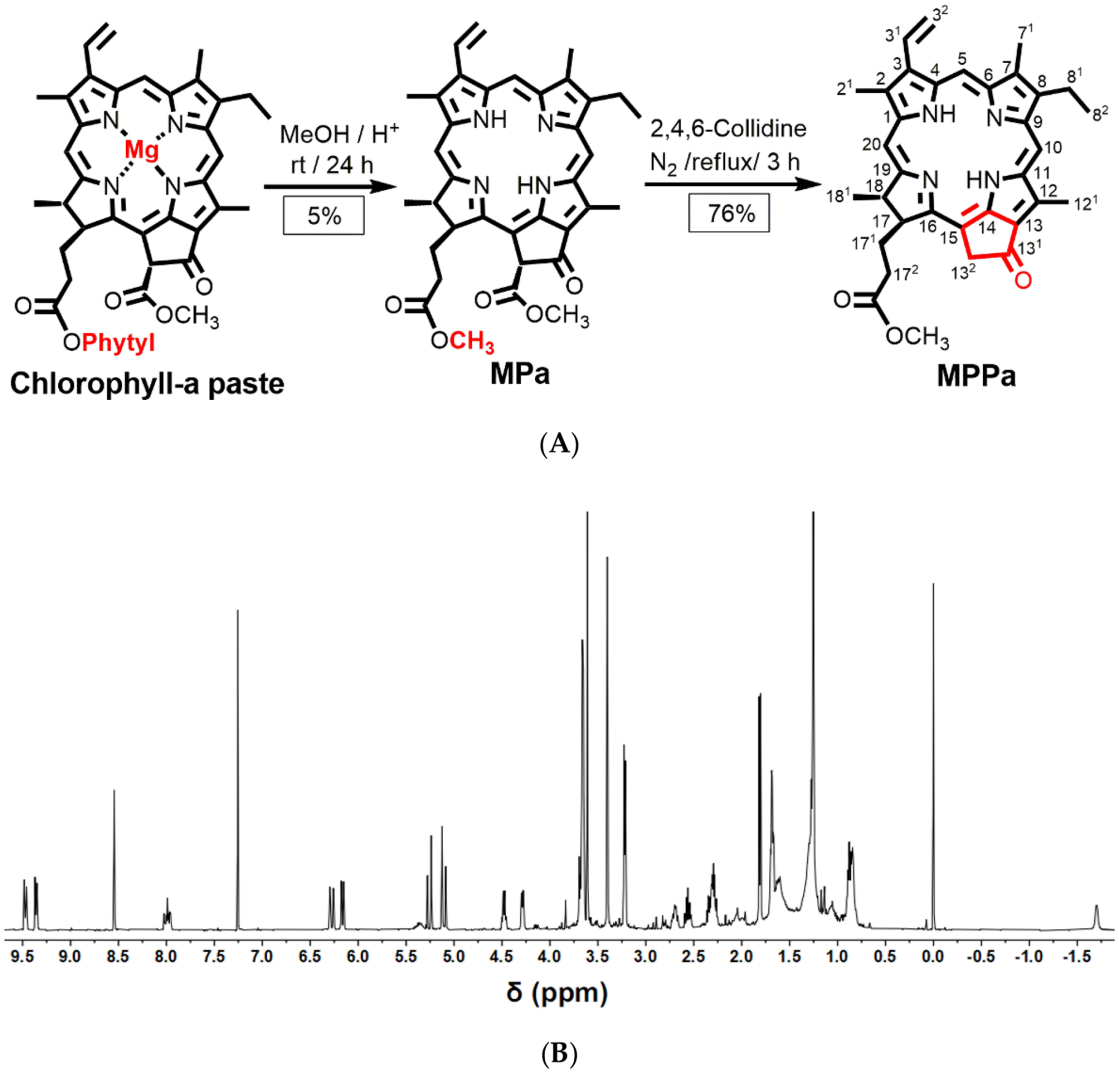
(**A**) Synthesis scheme of MPPa from chlorophyll-a with numbering, (**B**) ^1^H-NMR spectrum of MPPa (500 MHz, CDCl_3_, 25 °C, TMS).

Methyl pheophorbide-a (MPa) was extracted from chlorophyll-a paste according to a previously reported procedure (Fig. 1A)^30^. The synthesized MPa (1 g) was dissolved in 2,4,6-collidine (2,4,6-trimethylpyridine, 100 mL) and refluxed for 3 h, followed by cooling, evaporation of collidine, and washing with 2% HCl/MC (1:1). The obtained organic layer was evaporated and the residue was separated by column chromatography using 2% acetone/MC as the eluent to obtain pure MPPa.

### Preparation of MPPa-loaded SLNs

MPPa was loaded into SLN using a modified oil-in-water (O/W) emulsion method^31^. To prepare the O-phase, MPPa was added to the heated lipid at 10 °C above the lipid melting point. Subsequently, the O phase was added to the surfactant-dissolved W phase and homogenized using a polytron homogenizer (PT 3100) (Kinematica Instruments, Luzerne, Switzerland) at 1000 rpm to obtain an O/W emulsion. The obtained O/W emulsion was subjected to sonication to prepare SLN using a probe sonicator (Scientz-IID, Ningbo, China) at 300 W for 15 min with a 5 s pulse-on and a 5 s pulse-off period. The different compositions of MPPa-loaded SLN are shown in Table 1.

**Table 1:** Composition of MPPa-loaded SLNs.

Formulation	Drug (mg)	Lipid (mg)			Surfactant (mg)		
	MPPa	PA	SA	GMS	PX 188	PX 407	TW 80
F1	10	100			200		
F2	10		100		200		
F3	10			100	200		
F4	10			100		200	
F5	10			100			200
F6	10			100	400		
F7	10			300	200		
F8	10			300	400		
F9	10			500	200		
F10	10			500	400		

*MPPa* methyl pyropheophorbide-a, *PA* palmitic acid, *SA* stearic acid, *GMS* glycerol monostearate, *PX 188* poloxamer 188, *PX 407* poloxamer 407, *TW 80* Tween^®^ 80.

### NMR spectroscopy of MPPa

All ^1^H-NMR experiments were performed using a Varian spectrometer (500 MHz, CDCl_3_) at the Biohealth Products Research Center, Inje University^31,32^.

### Development of analytical method for MPPa

The MPPa concentration was determined using a UV–Vis spectrophotometer (S-3100, Scinco, Seoul, Korea) at ambient temperature^31,32^. To determine the maximum absorption wavelength of MPPa, the absorption spectrum was measured in the wavelength range 300–800 nm. The solvent that demonstrated the best characteristics for this method was MeOH. A standard stock solution was prepared by dissolving 2 mg of an accurate amount of MPPa in 20 mL of MeOH.

The standard stock solution was diluted with MeOH to obtain final concentrations of 1–20 ppm, and five-point linearity was determined. Standard solutions of different concentrations were prepared. Calibration curves and concentration versus absorbance units were constructed for each drug.

The precision of the test method was determined by performing an assay with six replicates of samples at test concentrations, and the relative standard deviation (RSD) of the assay results was calculated.

To study the accuracy of the method, recovery studies were performed by adding a known quantity of the standard to the pre-analyzed sample. The recovery was performed at 0%, 25%, and 100% levels, and the contents were measured from the respective UV–Vis absorption spectra.

### Determination of nanoparticle size, polydispersity index (PDI), and zeta potential for MPPa-loaded SLNs

The particle size and PDI of the prepared SLNs were determined at 25 °C by dynamic light scattering using a Zetasizer Nano ZS (Malvern Instruments Ltd., Worcestershire, Malvern, UK)^31–33^. The zeta potential of SLNs was estimated from the electrophoretic mobility of the particle surface using a Zetasizer Nano ZS. The samples were diluted 10 times with distilled water (DW) before the measurement. The instrument was equilibrated before each measurement. Each value reported is the average of three measurements.

### Determination of drug-loading capacity MPPa-loaded SLNs

The entrapment efficiency (EE) and loading amount (LA) of MPPa-loaded SLNs were determined by centrifugation^29^. SLN preparations were diluted 10 times to a final volume of 1 mL and then gently vortexed. The suspension was then centrifuged at 190 g at 4 °C for 1 h. The free drug concentration in the supernatant was analyzed using a UV–Vis spectrophotometer. EE and LA were calculated using Eqs. (1) and (2), respectively.

1 $$\mathrm{EE\, }\left(\mathrm{\%}\right)=\frac{\mathrm{Amount\, of\, total\, drug\, content}-\mathrm{Amount\, of\, free\, drug}}{\mathrm{Amount \,of\, total\, drug\, content}}\times 100,$$

2 $$\mathrm{LA\, }\left(\mathrm{\%}\right)=\frac{\mathrm{Amount\, of\, total\, drug\, content}-\mathrm{Amount \,of\, free\, drug}}{\left(\mathrm{Amount\, of\, total\, drug\, content}-\mathrm{Amount\, of\, free\, drug}\right)+\mathrm{Amount\, of\, lipid}}\times 100.$$

### In vitro MPPa release studies

An in vitro MPPa release study was performed using the dialysis-bag method. Dialysis bags (Spectrum Laboratories, Inc., Compton, CA, USA) with a molecular weight of 10 kDa were soaked in DW for 12 h before the experiment^34^. A predetermined amount of each test substance was soaked in dialysis bags and both ends were sealed using a string. Dialysis bags were immersed in 70 mL vials containing 50 mL of receptor medium (PBS, pH 7.4). The vials were then placed in a shaking incubator (JSSI-100 T, JS Research Inc., Gongju, Korea) and shaken at 100 rpm and 37 ± 0.5 °C. At predetermined time intervals (1, 2, 4, 8, 12, 24 and 48 h), aliquots of 1 mL were withdrawn from the vial, passed through 0.45 μm membrane filters (SFCA Syringe Filters, Corning Inc., NY, USA), and immediately analyzed using a UV–Vis spectrophotometer.

### The drug release kinetics models

To explain the mechanism of MPPa releases from the SLNs, the MPPa release profiles of the SLNs were analyzed with various models of release kinetics including zero-order, first-order, Higuchi, and Korsmeyer-Peppas models using Eqs. (3), (4), (5) and (6), respectively.

3 $${Q}_{t}={K}_{0}t+{C}_{0},$$

4 $$logC=log\, {C}_{0}-{K}_{t}/2.303,$$

5 $${Q}_{t}={K}_{H}\, t\,1/2,$$

6 $${Q}_{t}=K\,{t}^{n},$$

where Q_t_ is the amount of drug release at time t, Q_0_ is the initial amount of drug in formulations, K_0_, K_t_, K_H_ are release rate constants, C_0_ is the initial concentration of drug.

### Photostability studies

The photostability of MPPa in SLNs was determined by comparison with MPPa in 0.1% MeOH solution^29^. The photostability of MPPa was monitored by recording its absorption spectrum at 700 nm. Briefly, 20 mL of MPPa or MPPa-loaded SLNs in a 0.1% MeOH solution (4.0 ppm) was irradiated with a light-emitting diode (LED) at different time intervals (0, 10, 20, 30 and 40 min). MPPa was then extracted from the formulations by adding 1 mL hexane to melt the lipids, followed by vortexing. A 0.1% MeOH layer containing the extracted MPPa was filtered through 0.22 μm filters, and the UV–Vis spectrophotometer was measured.

### ^1^O_2_ Photogeneration

^1^O_2_ photogeneration study was determined using DPBF. DPBF, a selective ^1^O_2_ acceptor, is bleached upon reaction with ^1^O_2_, leading to a decrease in the intensity of the DPBF absorption band^31,32^. Each sample (1 μM) with 50 μM of DPBF in DMSO was used to evaluate photogeneration. The negative control (NC) and positive control (PC) contained 50 μM DPBF and 1 μM MB with 50 μM DPBF, respectively. All samples prepared in the dark were placed in a 48-well plate and covered with an aluminum foil. The plate was irradiated (2 J/cm^2^) with an LED (645‒710 nm) for 15 min. The absorbance of each sample was measured at 418 nm using a microplate reader (Synergy HTX; BioTek, Winooski, VT, USA).

The ^1^O_2_ quantum yields of MPPa and all formulations were determined using DPBF as a probe and MB as the standard with that calculation of Eq. (7)^35,36^.

7 $$ \phi_{\Delta } = \frac{{\phi^{{S_{\Delta } }} }}{{\gamma^{{S_{\Delta } }} }}\gamma_{\Delta } , $$

*ɸ*_Δ_: singlet oxygen quantum yield, *ɸ*^S^_Δ_: singlet oxygen quantum yield of MB equal to 0.596, *ɣ*^S^_Δ_: photodynamic activity of MB, and *ɣ*_Δ_: photodynamic activity of specimen.

### In vitro photoirritation study using human tumor cells

The anticancer efficacy of PDT with MPPa was evaluated by investigating the cytotoxic effects of each component of SLNs in tumor cell lines after photoirradiation^29,34^. Two cell lines (HeLa from human cervical carcinoma and A549 from human lung carcinoma) were seeded into 48-well plates at 2 × 10^4^ cells/well, and the number of cells was calculated using a hemocytometer. Prior to each experiment, the cells were incubated for 24 h at 37 ± 0.5 °C in a humidified atmosphere with 5% CO_2_. Various concentrations (1, 2.5, 5, and 10 μM) of each sample were then added to each well. After 24 h, the exposed cells were rinsed with sterile PBS and incubated with 200 µL/well of the growth medium. The cells were then irradiated (2 J/cm^2^) with LED at a distance of 20 cm for 15 min. The treated cells were incubated for 24 h at 37 ± 0.5 °C and 5% CO_2_ for the WST reduction experiment.

### Viability of cancer cells

Cytotoxicity was determined by measuring the dehydrogenase activity of viable keratinocytes at 24 h after incubation. Activity was determined after the incorporation of WST, as previously described^37^. Each cell line was treated with 100 µL/well of a 10% WST solution for 1 h. The WST concentration was measured by determining the optical density (OD) at 450 nm using a microplate reader.

Each experiment was conducted in at least three wells of a plate. After subtracting the blank OD from all raw data, the mean OD values ± standard deviations (SDs) were calculated using three measurements per test substance, and the percentage of cell viability relative to that of the NC was expressed using Eq. (8). The NC value was set at 100%.

8 $$\mathrm{Viability\, }\left(\mathrm{\%}\right) = \frac{{\mathrm{Mean\, OD}}_{\mathrm{treated}}}{{\mathrm{Mean \,OD}}_{\mathrm{control}}} \times 100$$

### Statistical analysis

Three independent experiments were performed for all analyses. The presented data (mean ± SD) were compared using a one-way analysis of variance and Student’s t-test. Statistical significance was set at p < 0.05.

## Results and discussion

### NMR spectroscopy of MPPa

The structure of MPPa was characterized by ^1^H-NMR spectroscopy. Figure 1B shows the ^1^H-NMR spectrum of MPPa. ^1^H-NMR (500 MHz, CDCl_3_, 25 °C, TMS): δ 9.48 (d, J = 12.0 Hz, 1H, 10H), 9.37 (d, J = 10.2 Hz, 1H, 5H), 8.54 (s, 1H, 20H), 8.03–7.95 (m, 1H, 3^1^H), 6.30 (d, J = 17.9 Hz, 1H, 3^2^H), 6.17 (d, J = 11.6 Hz, 1H, 3^2^H), 5.28 (d, J = 19.5 Hz, 1H, 13^2^H), 5.13 (d, J = 19.5 Hz, 1H, 13^2^H), 4.48 (m, 1H, 18H), 4.30 (m, 1H, 17H), 3.69 (m, 2H, 8^1^CH_2_), 3.66, 3.40, 3.23 (each s, 9H, CH_3_), 2.69 (m, 1H, 17^2^CH_2_), 2.29 (m, 2H 17^1^CH_2_), 2.04 (m, 1H, 17^2^CH_2_), 1.82 (d, J = 7.4 Hz, 3H, 18^1^CH_3_), 1.69 (t, J = 7.6 Hz, 3H, 8^2^CH_3_), − 1.70 (br, 2H, NH). Our results revealed that the peak for -OCH_3_ (13^4^H at 3.88 ppm as previously reported by us) in MPa disappeared after elimination of –COOCH_3_, and one proton signal of 13^2^ at 5.13 ppm appeared that confirmed the formation of reduced five-membered ring in MPPa^32^.

### Development of analytical method for MPPa

**Figure 2:**
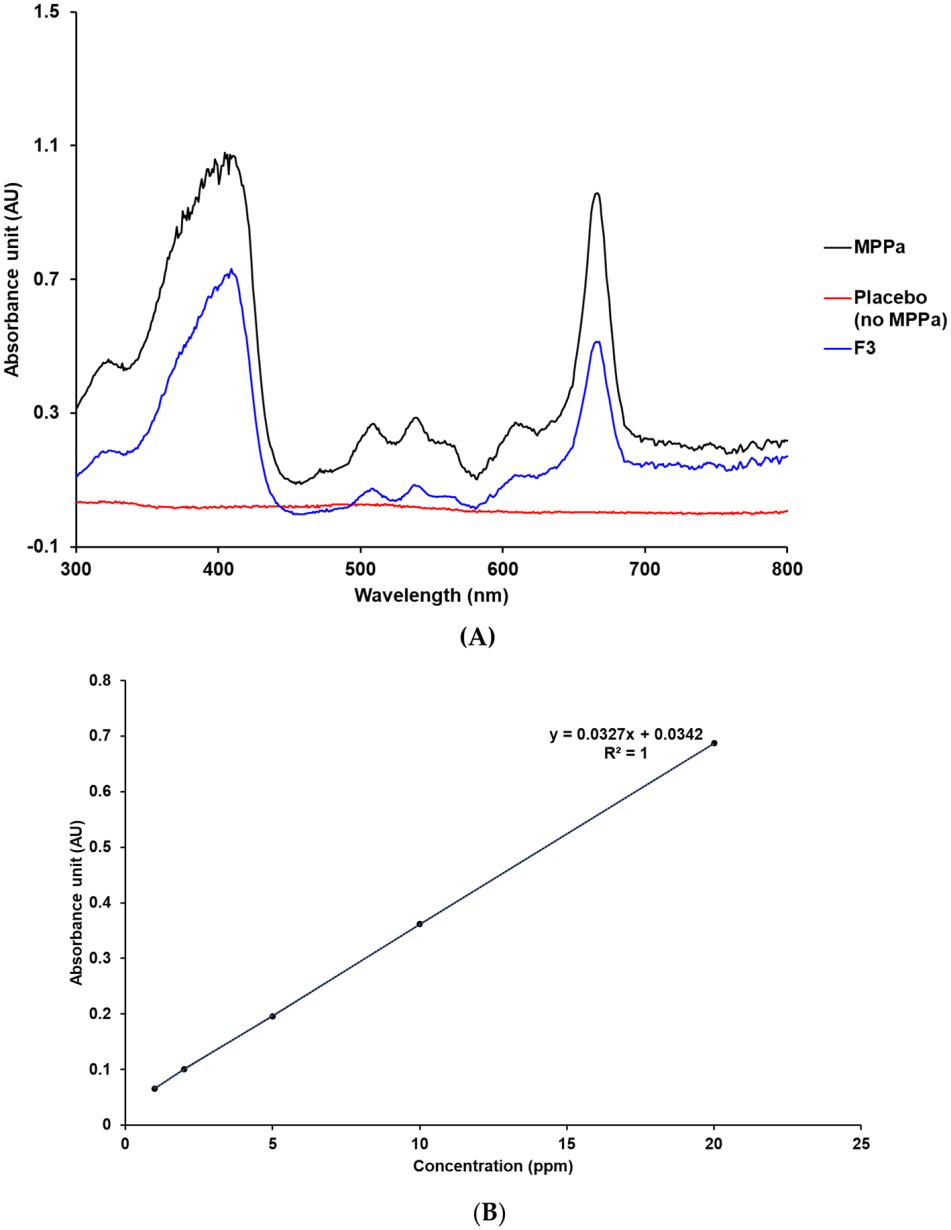
UV–Vis spectra and calibration curve of MPPa. (**A**) Specificity data of MPPa, placebo (no MPPa), and MPPa-loaded SLN F3 (MeOH, 25 °C). (**B**) Linearity data of MPPa standard stock solution in MeOH.

We obtained absorption spectra using a UV–Vis spectrophotometer to determine the specific absorption wavelength of MPPa (Fig. 2A). The specificity of MPPa was determined using MPPa standard stock solution, MPPa-loaded SLN, and placebo SLN (SLN without MPPa). The UV–Vis spectra of MPPa demonstrated that the maximum absorption wavelength was 667 nm. The placebo SLN had no interference spectra with that of MPPa. Therefore, we analyzed MPPa at 667 nm.

To calculate MPPa, we prepared a calibration curve using five standard stock solutions in a concentration range of 1–20 ppm. Figure 2B shows that the correlation coefficient obtained via the linear regression analysis was 1.

A precision study was performed to determine the closeness of the agreement for the same concentration with repeated measurements. The precision results are expressed as the RSD of repeatability. MPPa results demonstrated that the RSD (%) value of recovery was 1.06% (Table 2), which indicated the high precision of the proposed analytical method.

**Table 2:** Precision data obtained from the developed analysis of MPPa.

No	Recovery (%)
1	100.00
2	99.49
3	99.49
4	98.98
5	97.96
6	96.94
Average (%)	98.81
SD (%)	1.05
RSD (%)	1.06

We determined the accuracy via the developed analysis of MPPa to determine the closeness of agreement between the test results and a conventional true or accepted reference value. The recovery of MPPa was calculated and expressed as RSD. The RSD (%) values resulting from the accuracy were 1.26%, 0.42%, and 0.36%, respectively, as shown in Table 3.

**Table 3:** Accuracy data obtained from the developed analysis of MPPa.

Drug (ppm)	No	Recovery (%)	Average (%)	SD (%)	RSD (%)
1	1	100.00	98.48	1.24	1.26
	2	98.48			
	3	96.97			
5	1	100.00	99.49	0.42	0.42
	2	99.49			
	3	98.98			
20	1	100.00	99.56	0.36	0.36
	2	99.56			
	3	99.13			

### Particle characterization of MPPa-loaded SLNs for MPPa-loaded SLNs

**Figure 3:**
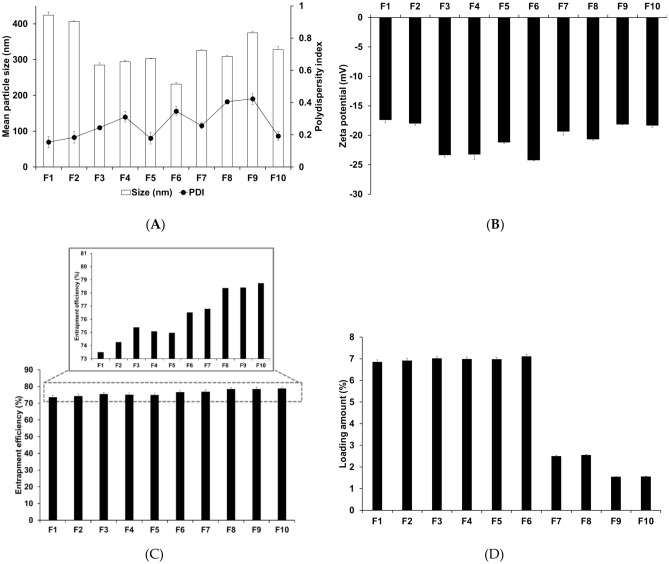
Particle characteristics. (**A**) Average particle size and PDI, (**B**) zeta potential, (**C**) Entrapment efficiency, and (**D**) loading amount of MPPa-loaded SLNs prepared using different materials. Results are expressed as the means ± standard deviations of three independent experiments (*n* = 3). PDI, polydispersity index.

Considering particle characterization, nanoparticle size, PDI, and zeta potential were determined to investigate the effects of different lipids and surfactants. Particle size is an important parameter in cancer therapy and is based on the EPR effect as a passive targeting strategy^26,38^. For nanoparticles, the zeta potential is important because it indicates the electric surface potential on the particles that ensures particle stability^18,39^. The results of particle size, PDI, and zeta potential demonstrated that all the formulations ranged from 231.37 to 424.07 nm in size, had a PDI of 0.15 to 0.42, and zeta potential of − 17.37 to − 24.20 mV, respectively, as shown in Fig. 3A,B. The particle size and zeta potential of formulations prepared using PA, SA, and GMS (F1, F2, and F3) were 424.07, 406.63, and 284.90 nm and − 17.37, − 17.97 and − 23.33 mV, respectively. This observation suggests that lipids with long carbon chains have a high affinity for MPPa, resulting in a small particle size and high zeta potential. The high affinity of lipids for MPPa could facilitate interaction with each other rather than the outer water phase^40–42^. The results of particle characterization for F3, F4, and F5, which were fabricated using different surfactants, demonstrated that F3 had the smallest size and highest zeta potential. This result suggests that a hydrophilic surfactant stabilizes the interface between O and W in O/W emulsions^39^. The high hydrophilic-lipophilic balance (HLB) of a hydrophilic surfactant is more stable than that of a relatively low HLB value. The HLB values of PX 188, PX 407, and TW 80 were 29, 22 and 15, respectively. Therefore, we selected GMS as a solid lipid and PX 188 as a surfactant to prepare SLN.

Considering the effects of lipid and surfactant concentrations on F3 and F6 to F10, the results of particle characterization demonstrated that an increase in the concentration of GMS as a lipid led to an increase in the particle size. For the surfactant, an increase in the concentration of PX 188 led to a decrease in the particle size. This observation suggests that the concentration effect of lipids is associated with the increased volume of the lipid matrix^23^. An increase in the concentration of surfactant causes the interface between O and W in the O/W emulsion to be effectively stabilized (increased zeta potential), and consequently reduces the particle size and enhances the particle stability^39^.

### Determination of the drug-loading capacity

Loading capacity (EE and LA) is a significant parameter in the formulation of lipid particle systems because it enhances the photostability of MPPa and avoids side-effects in the human body. Figure 3C,D show the EE and LA of MPPa-loaded SLNs. The EE and LA of all formulations were 73.49–78.73% and 1.54–7.11%, respectively. Among F1, F2, and F3, which were prepared using different lipids, F3 prepared from GMS showed the greatest loading capacity. This observation suggests that longer chain fatty acids have a high affinity for MPPa^43–45^, as mentioned in the particle characterization section. Regarding the effect of surfactants, F3 using PX 188 had a high amount of EE among F3, F4, and F5. This suggests that the high HLB value of the surfactant affected the stable dispersion of the MPPa-dissolved O phase into the W phase^43,46,47^. Regarding the effect of lipid and surfactant concentrations, an increase in the concentration of both increased EE owing to an increase in the volume of the lipid matrix and particle stability.

### In vitro MPPa release studies

**Figure 4:**
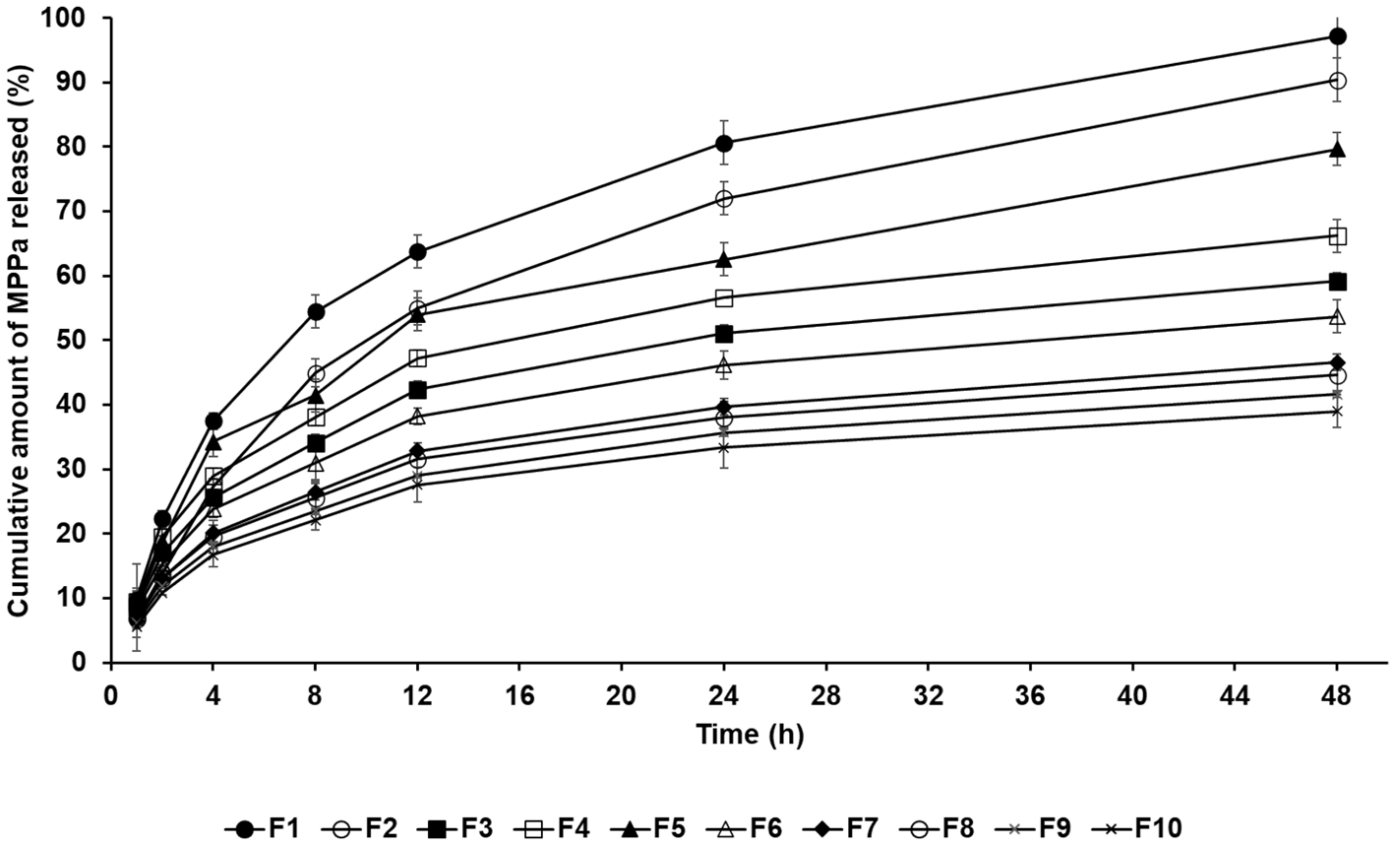
Cumulative percentage release profiles of MPPa from SLNs in the release medium, as determined using the dialysis bag method. Results are expressed as the means ± standard errors of three independent experiments (*n* = 3).

The release profile of MPPa-loaded SLNs was determined using the dialysis membrane method. The cumulative percentage release of MPPa-loaded SLNs was in the order F1 > F2 > F5 > F4 > F3 > F6 > F7 > F8 > F9 > F10. Among the formulations of F1 to F6, this order was the same as the particle size and reverse of the order of zeta potential (low stability), as shown in Fig. 3A and B. Thus, the large particle size and low stability of SLNs induced high drug release from SLNs after 48 h. The MPPa release from SLN exhibited a sustained release profile, as shown in Fig. 4. The sustained release of MPPa was biphasic, with a relatively burst and delayed release. The relatively burst release of F1–F6 and F7–F10 was observed over 12 and 8 h, respectively, followed by a sustained release for 48 h. This observation suggests that the relatively burst release is attributed to the adhesive MPPa on the particle surface (shell), whereas the sustained release is owing to the MPPa encapsulated into the particle core^23^. Concerning the short burst release of F7–F10, formulations with a high loading capacity are encapsulated in the particle core rather than the shell, which leads to a delayed release^23^. In this sense, the order of MPPa release was exactly the reverse of the order of EE, as shown in Fig. 3C.

### The drug release kinetics models

The MPPa release results were analyzed using release kinetics models (zero order, first order, Higuchi, and Korsmeyer-Peppas). Table 4 demonstrates that Higuchi model is the highest correlation coefficients (R^2^) values. This suggests that MPPa was homogeneously loaded in entire lipid matrix of SLN. In this regard, MPPa release was dominated by diffusion and dissolution^48,49^.

**Table 4:** Correlation coefficients (R^2^) values for the in vitro release profiles fitted with multiple drug-release kinetics.

Formulations	Correlation coefficient (R^2^) values of drug-release kinetics			
	Zero order	First order	Higuchi	Korsmeyer-Peppas
F1	0.778	0.993	0.956	0.695
F2	0.830	0.988	0.971	0.783
F3	0.739	0.837	0.940	0.675
F4	0.740	0.859	0.940	0.661
F5	0.778	0.934	0.953	0.691
F6	0.739	0.825	0.940	0.678
F7	0.748	0.818	0.944	0.709
F8	0.745	0.812	0.944	0.694
F9	0.752	0.812	0.947	0.712
F10	0.749	0.804	0.945	0.729

### Photostability studies

**Figure 5:**
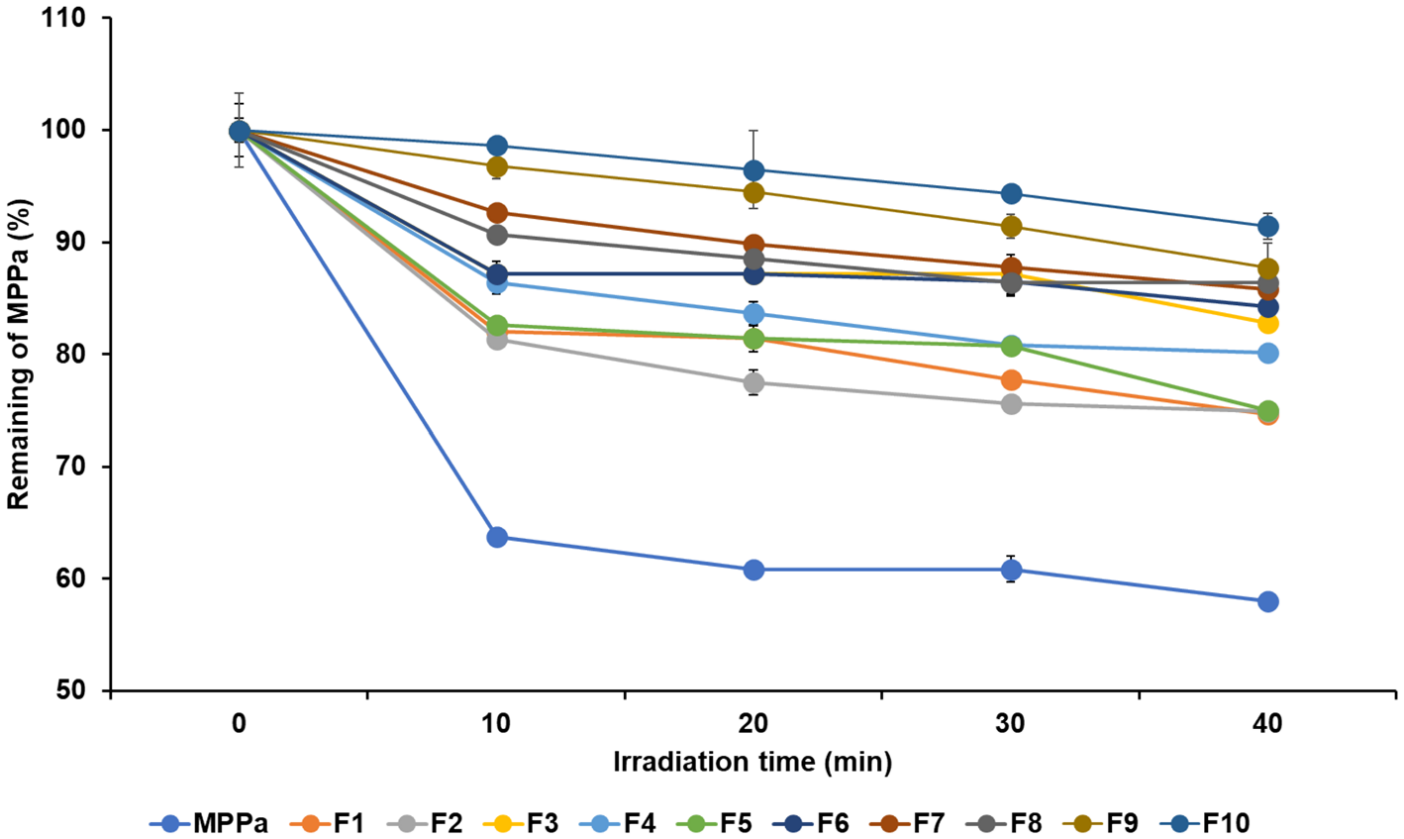
Photostability test using percentage of non-degraded MPPa from MPPa solution and MPPa-loaded SLNs before and after irradiation with an LED of 2 J/cm^2^ for different time intervals of 0, 10, 20, 30, and 40 min. Results are expressed as means ± standard deviations of three independent experiments (*n* = 3).

In PDT, the photostability of the PS is important because it is closely related to its pharmacological effect. Our results revealed that all formulations improved the photostability of MPPa (Fig. 5). The order of photostability after 40 min of irradiation was F10 > F9 > F8 > F7 > F6 > F3 > F4 > F5 > F2 > F1 > MPPa; this order was the exact reverse of that observed for the release profile of MPPa from SLNs shown in Fig. 4. The remaining concentration of MPPa from the MPPa solution was 57.97% and that from the formulations ranged from 74.67% to 91.43%. Thus, a solid lipid, as the main ingredient of SLN, physically prevents any interruption from environmental factors and maintains MPPa for a relatively long time^22,23,50^. The concentration effects of lipids and surfactants demonstrated that F7–F10 highly improved the photostability of MPPa probably because formulations with high loading capacity protect MPPa from environmental factors.

### ^1^O_2_ photogeneration

**Figure 6:**
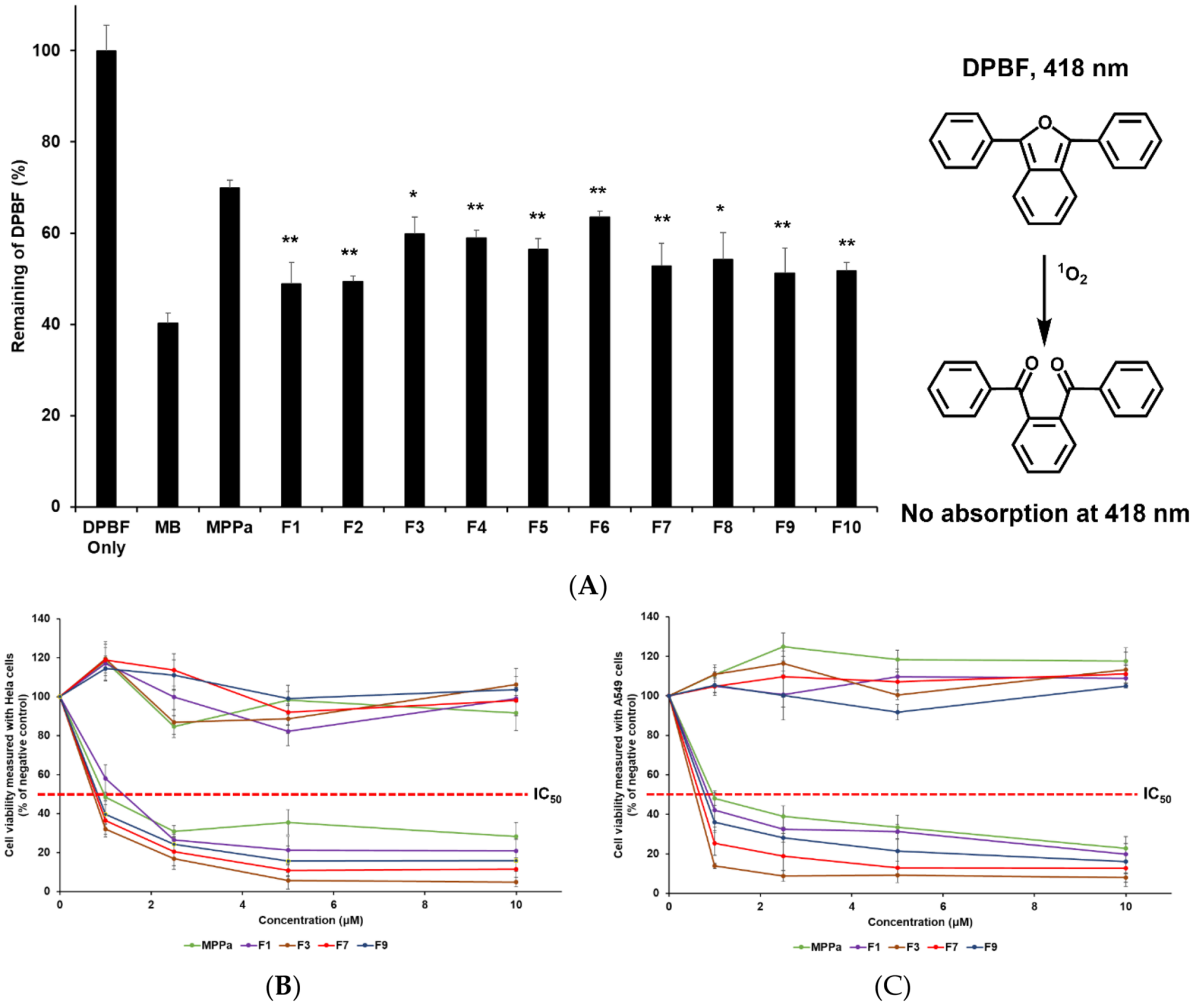
Photodynamic therapy (PDT) efficacy. Non-biological efficacy of (**A**) DPBF (50 μM in DMSO) absorbance decay (%) for the ^1^O_2_ photogeneration efficacy of MPPa with/without SLNs at 418 nm after photoirradiation (total light dose 2 J/cm^2^; irradiation time 15 min). Statistical significance of the difference in DPBF between MPPa solution and the formulations is indicated by either a single asterisk (*p* < 0.05) or double asterisks (*p* < 0.01). Biological efficacy for (**B**) HeLa cell line and (**C**) A549 cell line treated with MPPa solution, F1, F3, F7, and F9. The cell viability was measured using the WST assay. Results are expressed as means ± standard deviations of three independent experiments (*n* = 3).

The efficacy of PDT was investigated using the DPBF assay where DPBF reacts with ^1^O_2_, which decreases the intensity of the DPBF absorption band^51,52^. We performed the DPBF assay with photoirradiation of the MPPa solution and all formulations to detect the generated ^1^O_2_, as shown in Fig. 6A. MB, a standard ^1^O_2_ sensitizer, was used as the PC. Additionally, high ^1^O_2_ quantum yields can promise high anti-cancer efficacy in PDT. The ^1^O_2_ quantum yields of MPPa and all formulations were measured and compared using DPBF as a probe and MB as the standard. The ^1^O_2_ quantum yield was calculated according to the literature^35,36^. DPBF results demonstrated that all formulations of SLNs exhibited better ^1^O_2_ photogeneration than the MPPa solution. This indicates that the PDT efficacy of all test substances was lower than that of MB. The MPPa-loaded SLNs improved the PDT efficacy of MPPa as compared to the MPPa solution. Thus, the aggregation of MPPa is prevented by applying SLN^43,46^. In addition, among the formulations of F1–F6, the order of the ^1^O_2_ photogeneration efficiency (^1^O_2_ quantum yields) was F1 (0.510) > F2 (0.505) > F5 (0.435) > F4 (0.410) > F3 (0.401) > F6 (0.365), which is the same as that observed for the release profiles in Fig. 4 and exactly opposite of the order reported for zeta potential and photostability in Figs. 3B and 5, respectively. Therefore, highly stable formulations afforded relatively low PDT efficacy among MPPa-loaded SLNs. The reason for this might be the environmental protective effect of the SLN system^22,23^.

### In vitro photoirritation studies

Photocytotoxicity was evaluated as PDT efficiency using HeLa cells from human cervical carcinoma and A549 cells from human lung epithelial carcinoma. We selected F1, F3, F7, and F9 among the formulations based on particle size and EE results. We conducted cytotoxicity assay to determine the safety of the formulations because the PDT mechanism for ^1^O_2_ generation occurs under light^2,3,44^. Figure 6B and C shows the photocytotoxicity against HeLa and A549 cells. Under dark conditions, the viability of HeLa and A549 cells treated with 10 μM of the test substances ranged from 91.76 to 106.31% and 105.00 to 117.65%, respectively. These results revealed that MPPa and MPPa-loaded SLNs were not cytotoxic to HeLa and A549 cell lines.

Regarding the photocytotoxicity of MPPa, all test substances demonstrated anticancer effects depending on the cell type and concentration of MPPa. Four different concentrations of each test substance (1, 2.5, 5 and 10 μM) were used to estimate the 50% inhibitory concentration values (IC_50_). The results of IC_50_ are summarized in Table 5. The order of PDT efficacy was the same as the order of zeta potential and the opposite of the order of particle size as following: F3 (0.74 μM) > F7 (0.79 μM) > F9 (0.83 μM) > MPPa (0.97 μM) > F1 (1.38 μM) in HeLa cells; F3 (0.58 μM) > F7 (0.67 μM) > F9 (0.78 μM) > F1 (0.89 μM) > MPPa (0.96 μM) in A549 cells. Therefore, a small particle size with low aggregation (high zeta potential) results in better PDT efficiency. Regarding the effect of different lipids, F3 prepared using GMS showed high anticancer effects against both cell lines as compared with F1 prepared using PA. Moreover, although F7 and F9 had a higher MPPa loading capacity than F3, the PDT efficacy of F3 was higher than that of F7 and F9. Considering that the particle size of F3 was smaller than that of F7 and F9, the anticancer effects via PDT efficacy dominated the particle size rather than the drug-loading capacity^3,53,54^. In addition, the order of PDT efficacy was the same as that of release, except for F1. This is because the formulation with large particle size showed low zeta potential as a stability parameter that is affected by both high particle aggregation and fast drug release, as mentioned in the sections on particle size and drug release. Thus, F3 is a promising formulation for cancer treatment based on the EPR effect strategy among the tested substances.

**Table 5:** IC_50_ (μM) values against HeLa or A549 cells, particle size, zeta potential, and entrapment efficiency (EE) of MPPa solution, F1, F3, F7, and F9.

	Hela (μM)	A549 (μM)	Particle size (nm)	Zeta potential (mV)	EE (%)
MPPa	0.97	0.96	N/A	N/A	N/A
F1	1.38	0.86	424.07 ± 8.63	− 17.37 ± 0.50	73.49 ± 1.20
F3	0.74	0.58	284.90 ± 6.26	− 23.33 ± 0.40	75.38 ± 1.19
F7	0.79	0.67	325.77 ± 1.62	− 19.33 ± 0.60	76.78 ± 1.21
F9	0.83	0.78	374.50 ± 5.22	− 18.13 ± 0.09	78.41 ± 1.21

*N/A* not applicable.

## Conclusion

In this study, we attempted to synthesize MPPa and fabricate MPPa-loaded SLNs as a promising cancer treatment for PDT to improve the photostability and pharmacological effects. ^1^H-NMR results showed that all proton signals were assigned, indicating the successful synthesis of MPPa. All MPPa-loaded SLNs displayed highly enhanced photostability and ^1^O_2_ photogeneration compared with free MPPa. In terms of PDT efficacy, our SLNs showed better anticancer effects than free MPPa against HeLa and A549 cells. In addition, the cytotoxicity study was performed under dark and light conditions, which ensured that the normal state of MPPa and MPPa-loaded SLNs was safe unless otherwise irradiated. Among F1, F3, F7 and F9 SLNs, use of lipid with longer carbon chain (GMS) generated smaller particle sizes of SLNs. In addition, a decrease of the lipid (GMS) concentration (among F3, F7 and F9) is important to decrease the particle size which induced an increase of the stability (increase of the zeta potential). Finally, F3 SLNs displayed the highest PDT efficiency against both cell lines, which might be attributed to the smallest particle size as well as the highest stability and loading amount even though the lower ^1^O_2_ photogeneration and entrapment efficiency. Therefore, we can make a conclusion that the anticancer efficacy of MPPa-loaded SLNs was dominated by particle size and stability rather than entrapment efficiency. Thus, these results showed that MPPa-loaded SLNs are promising anticancer agents for PDT.

## Data Availability

The datasets used and/or analysed during the current study available from the corresponding author on reasonable request.

## Abbreviations

DPBF: 1,3-Diphenylisobenzofuran
EE: Entrapment efficiency
EPR: Enhanced permeability and retention
FTIR: Fourier transform infrared spectroscopy
HLB: Hydrophilic-lipophilic balance
LA: Loading amount
LED: Light-emitting diode
MPPA: Methyl pyropheophorbide-a
NMR: Nuclear magnetic resonance
PDT: Photodynamic therapy
PS: Photosensitizer
RSD: Relative standard deviation
SLN: Solid lipid nanoparticle
